# Scoping review to assess online guidance about poultry farm biosecurity for farmers in the UK

**DOI:** 10.1002/vetr.5775

**Published:** 2025-12-04

**Authors:** Eve Houghton, Ivo Syndicus, Paniz Hosseini, Timothy Borthwick, James L. N. Wood, Pablo Alarcon

**Affiliations:** ^1^ Veterinary Epidemiology Economics & Public Health Group University of London Hertfordshire UK; ^2^ Department of Veterinary Medicine University of Cambridge Cambridge UK; ^3^ Independent Scholar

## Abstract

**Background:**

Biosecurity is the primary defence against diseases such as avian influenza (AI) for poultry farms. This research reviews online resources to assess the availability and topics shaping biosecurity advice for poultry farmers.

**Methods:**

A rapid scoping review examined biosecurity guidelines available to UK poultry farmers on 10 websites (www.gov.uk; www.gov.scot; www.gov.wales; www.bfrepa.co.uk; www.nfuonline.com; www.britishpoultry.org.uk; www.redtractor.org.uk; www.bvpa.co.uk; www.bva.co.uk; www.britisheggindustrycouncil.com). Webpages were analysed against key criteria, including topics and levels of detail.

**Results:**

Ultimately, 174 webpages were analysed. Government websites housed many pages relevant to poultry biosecurity, while assurer and trade union sites contained fewer. There was a broad consensus across websites on the topics covered, with hygiene and administrative guidance included on the largest number of webpages. Most webpages contained only limited or some relevant details, often repeating key messages rather than offering comprehensive implementation guidance. Pages with extensive detail were linked to regularly and appeared early in search results.

**Limitations:**

Northern Ireland's government website was not reviewed.

**Conclusion:**

Many webpages advising on poultry farm biosecurity exist. They often highlighted the importance of hygiene measures and administrative actions. However, the lack of detailed implementation guidance may present challenges for farmers seeking to learn how to implement good biosecurity.

## INTRODUCTION

Since 2016, the UK has experienced large outbreaks of highly pathogenic avian influenza (AI).^1^ In 2022‒2023, the period with the most outbreaks, the virus was detected in 206 poultry premises across England (*n* = 160), Scotland (*n* = 38) and Wales (*n* = 8), resulting in millions of dead and culled birds.^2^ Infection in poultry farms has been attributed to high levels of wild bird infections, with 1876 wild birds testing positive in 2022,^3^ and little evidence of lateral spread between farms. In response, the UK government implemented measures to mitigate the risk of disease incursions, including a policy requiring all poultry to be kept indoors, and drew on powers granted by the Animal Health Act 1981^4^ to introduce AI protection zones in October 2022. These zones and associated orders have been periodically phased in and out since, with the most recent zones introduced in December 2024.^5^ In the UK, biosecurity is considered the primary—and often only—disease prevention strategy for combatting routes of disease incursion. However, challenges remain in ensuring biosecurity measures are being effectively implemented on farms. Research suggests that farming practices are informed by differing stakeholder perceptions of risk, responsibility and control, influencing the types and extent to which biosecurity measures are applied.^6^

To improve biosecurity applications on poultry farms, it is crucial to understand how biosecurity measures are currently implemented and what information farmers have access to that informs their priorities and understanding of biosecurity. Research regarding how information is accessed and what kinds of information are trusted among livestock keepers in the UK is limited and has predominantly been focused on sheep and pig farms^7^ or backyard poultry.^8^ In these studies, the sources of information informing animal health and disease management decisions differed greatly across farms raising different livestock. Thus, there is cause to examine the commercial poultry sector more closely to understand the unique situation on poultry farms.

There is also a need to understand the extent to which biosecurity is defined, communicated and applied through government and industry. Thus, the aim of this study was to conduct a scoping review of webpages from the government and key organisations offering biosecurity information to poultry farmers. Analysis of webpage content helped identify how stakeholders define biosecurity through the topics covered by biosecurity‐related pages. Following this, the study analysed the level of detail provided against each biosecurity topic and the number of links a farmer would need to follow to access it.

## MATERIALS AND METHODS

### Selection of webpages to review

**FIGURE 1:**
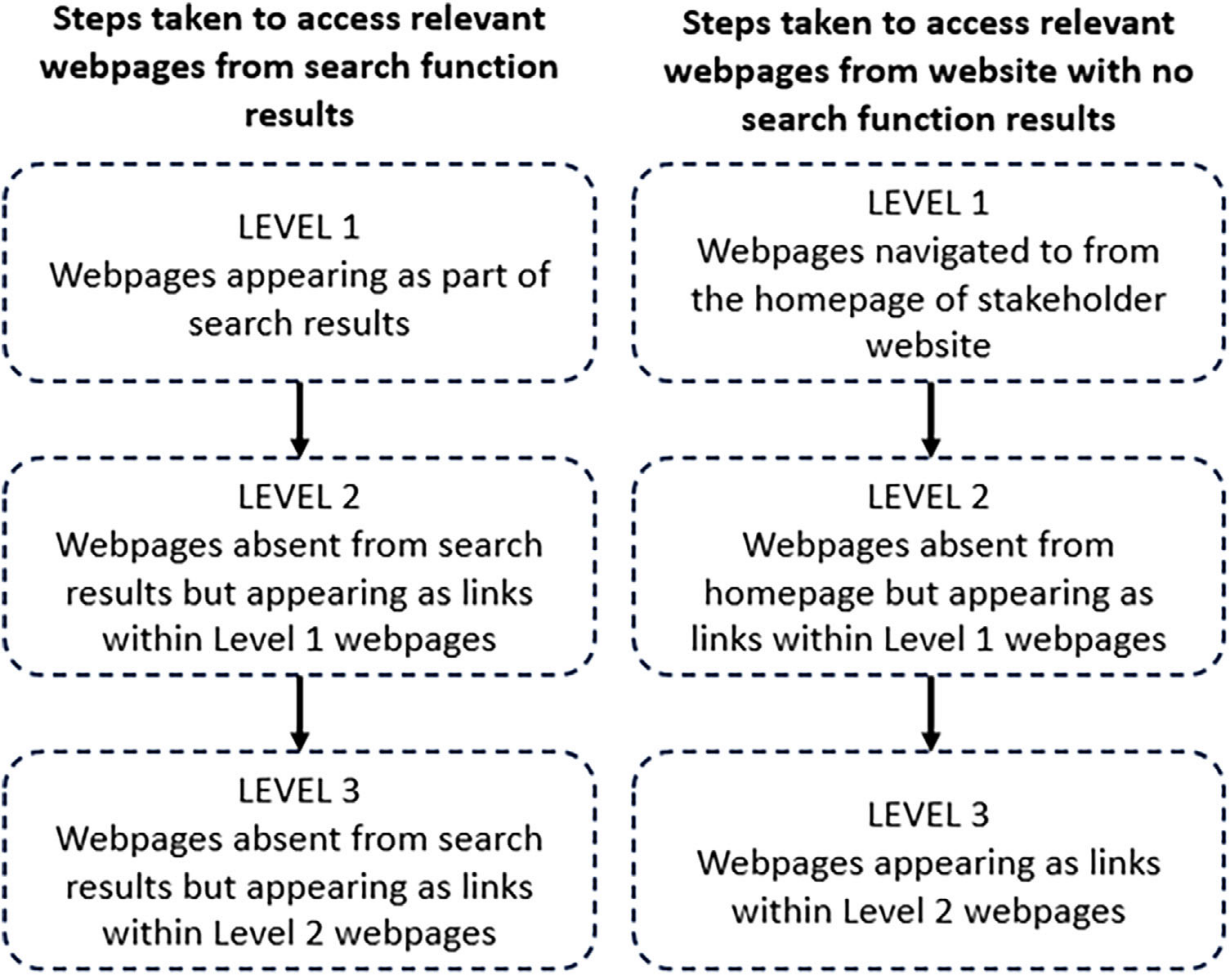
Steps taken to access relevant webpages.

Webpages were selected through a three‐stage approach (Figure 1).

#### Stage 1—Website identification

The review included websites of 10 stakeholders associated with commercial poultry farming in the UK: the UK Government, the Scottish Government, the Welsh Government, the British Free Range Egg Producers Association (BFREPA), the National Farmers’ Union (NFU), the British Poultry Council (BPC), Red Tractor and Red Tractor Assurance, the British Veterinary Poultry Association (BVPA), the British Veterinary Association (BVA) and the British Egg Industry Council (BEIC). These stakeholders were identified through consultation with the Animal and Plant Health Agency and academics working in animal health at the Royal Veterinary College and the University of Cambridge. Throughout this paper, ‘websites’ refers to the domain name housing multiple ‘webpages’, which are referred to as single specific pages found within a domain.

As a rapid review, not every relevant website could be included. Industry websites that focused on Great Britain were prioritised, excluding the government of Northern Ireland's website. We did not include all specialised websites (e.g., Game Farmers’ Association), which may have revealed more targeted information available to certain poultry farmers.

#### Stage 2—Webpage identification

Commencing 9 April 2024 and concluding on 7 September 2024, website searches were conducted using the phrase ‘biosecurity poultry’. On websites dedicated to poultry, the search was ‘biosecurity’ only. Two of the 10 websites did not have search functions (BEIC and BVPA) and one website (BFREPA) generated no search results. In these cases, websites were navigated to seek relevant information based on page titles.

From the search results, a list of criteria was used to identify pages for analysis. Exclusion took place over three rounds (Table 1). The first round was manually conducted by a single researcher, checking search result webpage titles for relevance. The URLs of relevant, unique webpages were saved for review in exclusion rounds 2 and 3.

**TABLE 1:** Exclusion criteria across three rounds of webpage review to narrow search results to retain only relevant content for data extraction and analysis.

Exclusion round	Exclusion criteria
1	Based on the titles, webpages were excluded where they did not fulfil one or more of the following: Relate to poultry, wild birds, farm disease incursions or poultry farms in the UK. Include explicit guidance or policy/legislation relating to biosecurity and/or AI. Duplicate pages were removed.Notices of AI cases in the UK were excluded.
2	Each webpage was opened and based on a skim read, items were excluded when they were: Unrelated to the UK. Not explicitly framed as relevant for biosecurity. Not related to poultry farms. Reports about AI status in the UK, but not guidance. Outdated rules/guidelines since revised. Duplicate links remaining from search results.
3	Based on an in‐depth read of each webpage, items were only excluded in the third round when it was found they should have been excluded earlier.

Abbreviation: AI, avian influenza.

#### Stage 3—Webpage finalisation

During round 3, relevant onward links from each webpage were added to the final list of webpages. Unique pages were added and recorded as ‘level 2’ or ‘level 3’, indicating how many clicks were needed to reach the page. If links led to external sources, the content was analysed as part of the website on which the link was found, but its external source affiliation was noted.

### Data extraction

Data extraction captured the topics each page mentioned in relation to poultry biosecurity; the amount of detail provided (Table 3); whether the content was about AI and whether the webpage targeted specific farm types (see Supporting Information for full list of extracted data).

A list of biosecurity topics was generated during the third stage of the exclusion process. This list was also informed by findings from a previous stage of this project that explored the implementation of biosecurity measures on UK farms.^6^ The list was developed iteratively over the course of data extraction. This resulted in a final list of 35 topics that defined poultry farm biosecurity guidelines across the 10 websites reviewed. Once finalised, topics were organised into topic groups: administration, farm infrastructure and areas, hygiene, livestock management, organisation of inputs and wild animals (Table 2).

**TABLE 2:** Topic groups and topics relating to biosecurity captured for every webpage.

Topic group	Group definition	Topic	Topic definition
Farm admin	Any topic that involves paperwork as the main activity such as record keeping, licensing and farm registration. This theme also includes people management as a step associated with biosecurity, such as training staff.	Licensing	Page specifies the need for licenses as biosecurity measure and guidance on how to apply for them, for example, license to move poultry when in an avian influenza prevention zone
		Record keeping: birds	Page suggests that keeping records about the poultry on the farm is relevant to biosecurity, including production records; mortality; water and feed consumption; medicine use
		Record keeping: visitors	Page suggests that farms should register with the government as part of good biosecurity practice
		Reduce/restrict site access (at boundary and within farm)	Page suggests that access to the farm and specific buildings within it should be limited (no mention of physical interventions)
		Registration	Page suggests the need for farms to report signs of disease (to veterinarians, APHA or other government links)
		Reporting disease	Page promotes staff training as an aspect of attaining farm biosecurity
		Train/direct staff	Page suggests that a record of visitors should be kept, which may include recording vehicle entry
Farm infrastructure and areas	Any topic that involves physical buildings or spatial delineation of areas within the farm or signifying its boundary.	Dedicated hard area for vehicles	Page suggests that vehicles should have hard area for parking or cleaning
		Farm boundary	Page suggests that biosecurity efforts should include fencing or a clear division between on‐farm and off‐farm areas with a physical boundary or entry
		Farm building position and maintenance	Page suggests maintaining farm buildings and the importance of where they are located as relevant to biosecurity
		Restrict poultry access to waterways and ponds	Page suggests that farmers should restrict bird access to ponds or waterways through physical containment of birds or fencing/netting of water areas
		Step‐over/hygiene barrier	Page suggests that step over or hygiene barriers should be in place at points within the farm
		Ventilated livestock housing	Page suggests that having ventilation is relevant to biosecurity
		Visitor signage	Page suggests having biosecurity signage and site guidance for visitors
Hygiene	Any topic focusing on cleaning, disinfection or practices to maintain hygiene, including across the farm site, farm equipment, vehicles and people.	Cleaning site/equipment	Page suggests *what* should be cleaned and/or disinfected such as the farm, certain areas or equipment
		Clothing change	Page suggests that clothes should be *changed* when entering or moving between buildings on the farm
		Keep farm areas tidy	Page suggests keeping farm areas tidy, for example, removing weeds and not leaving equipment lying around
		PPE (using that term)	Page suggests using PPE as part of biosecurity measures on farms, using the term PPE rather than the specific equipment, for example, goggles, gloves, etc.
		Footwear change/cleaning/foot dips	Page suggests footwear management as a biosecurity practice, for example, footwear changes, cleaning, boot covers and foot dips
		Specific cleaning equipment/strategy (e.g., scrubbing)	Page suggests *how* cleaning should be performed, for example, promoting the use of particular equipment such as a power hose or using a specific approach such as scrubbing
		Sunlight as disinfectant	Page suggests that sunlight can work to disinfect farm items or areas
		Transporting/vehicle hygiene	Page suggests that vehicles within or entering the farm should be cleaned
		Washing/showering	Page suggests the need for human hygiene, which may include suggestions for particular washing practices (e.g., hand washing or disinfection) or facilities required for this
		Waste management	Page suggests that waste from the farm (including manure, carcasses, waste water, soiled bedding) should be handled in particular ways
Livestock management	Any topic describing how poultry (or any other animals on the poultry farm) should be handled, kept or managed in relation to disease.	Culling	Page suggests that culling may be involved as part of biosecurity process or response to disease outbreaks
		Disease surveillance	Page suggests that farm managers or workers should look for signs of disease
		Game bird management	Page suggests importance or restrictions relevant to game bird management and disease
		Keeping birds inside	Page suggests that birds should be kept inside as part of biosecurity efforts or rules relating to that when in prevention zones
		Medical treatments	Page suggests that treating birds with medicines is relevant to biosecurity (often not AI related)
		Separation of new stock, different flocks or infected birds	Page suggests that different breeds, flocks, species or infected birds should be kept separately or quarantined
		Vaccination (not just against AI)	Page suggests the use of vaccination
Organisation of inputs	Topics describing input management in relation to biosecurity efforts.	Bedding management (clean)	Page suggests fresh bedding should be handled or stored in a certain way as part of biosecurity effort
		Food and water sourcing, covering and containment	Page suggests specific food and water sourcing or suggests how it should be kept on the farm as part of biosecurity effort
Wild animals	Topics describing need for awareness or specific responses to wild animals in relation to biosecurity efforts.	Vermin monitoring/control	Page suggests that controlling vermin including rats and mice should be done as part of biosecurity effort and any suggestions for how to manage them
		Wild bird monitoring/management	Page suggests that wild birds should be considered relevant to biosecurity and any suggestions for how to manage them

Abbreviations: AI, avian influenza; APHA, Animal and Plant Health Agency; PPE, personal protective equipment.

### Content analysis

Content analysis involved a combination of conceptual analysis and quantification of the data that were extracted from each page. Identifying the topics that each page covered was a central part of the process, followed by an analytical review of each topic and the frequency with which it occurred on each website.

Timing impacted the granularity of analytical categories applied to the data. For instance, webpages covering multiple biosecurity topics were assigned a single level of detail, which might have masked differences between level of detail available on specific topics.

The dynamic nature of websites means that some content, such as the website of the BPC, changed after the review was complete, removing some webpages and changing the pathways to information used during this research.

## RESULTS

### Webpage inclusion and publication date

From across 10 stakeholder websites, 174 webpages were analysed. The websites with the largest number of relevant webpages were the UK (*n* = 46), Scottish (*n* = 34) and Welsh (*n* = 32) governments. The website with the fewest relevant webpages was the BEIC (*n* = 1) (Table 4).

**FIGURE 2:**
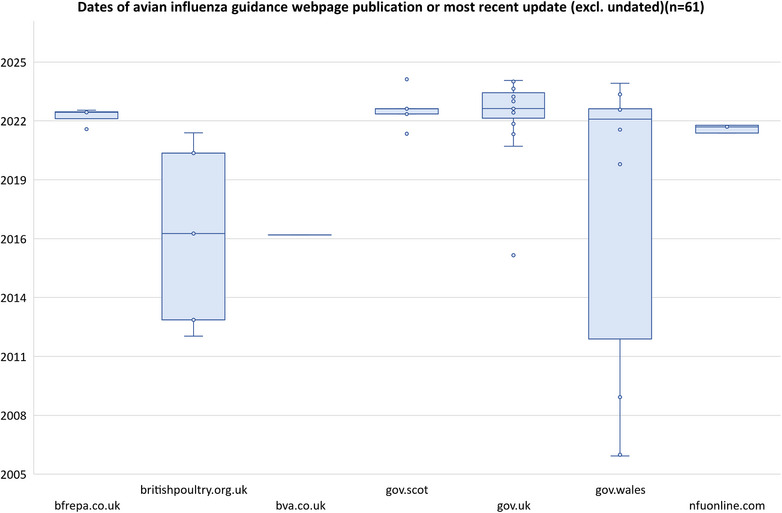
Dates of avian influenza guidance webpage publication or most recent update (excluding undated).

Of the 174 webpages, 133 included publication dates. These ranged from July 2002 to October 2024. Webpages dedicated to information on AI (*n* = 61) were published or updated between November 2006 and April 2024 (Table 5 and Figure 2), with most published within the last 4 years or updated within that time (*n* = 51). However, two websites contained AI advice on webpages that were over 10 years old.

### Biosecurity topics

**FIGURE 3:**
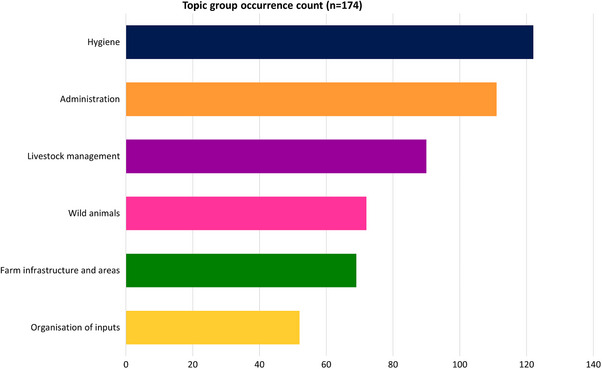
Topic group occurrence count.

Across the webpages reviewed, commercial poultry biosecurity guidance was made up of 35 topics. The topics fitted into six broader topic groups: administration, farm infrastructure and areas, hygiene, livestock management, organisation of inputs and wild animals (definitions available in Supporting Information), with some topic groups addressed on webpages more than others (Figure 3). The topics revealed a definition of biosecurity that is inclusive of not only on‐farm infrastructure and practices, but also farm surroundings, wildlife and disease reporting activities. Hygiene was the topic group that the largest number of webpages addressed. This included topics relating to farm, human and vehicle hygiene activities and waste management. Administration was the second most frequently included topic group. This included webpages that promoted activities that would not directly prevent disease but help veterinarians and authorities respond to outbreaks, such as record keeping.

**FIGURE 4:**
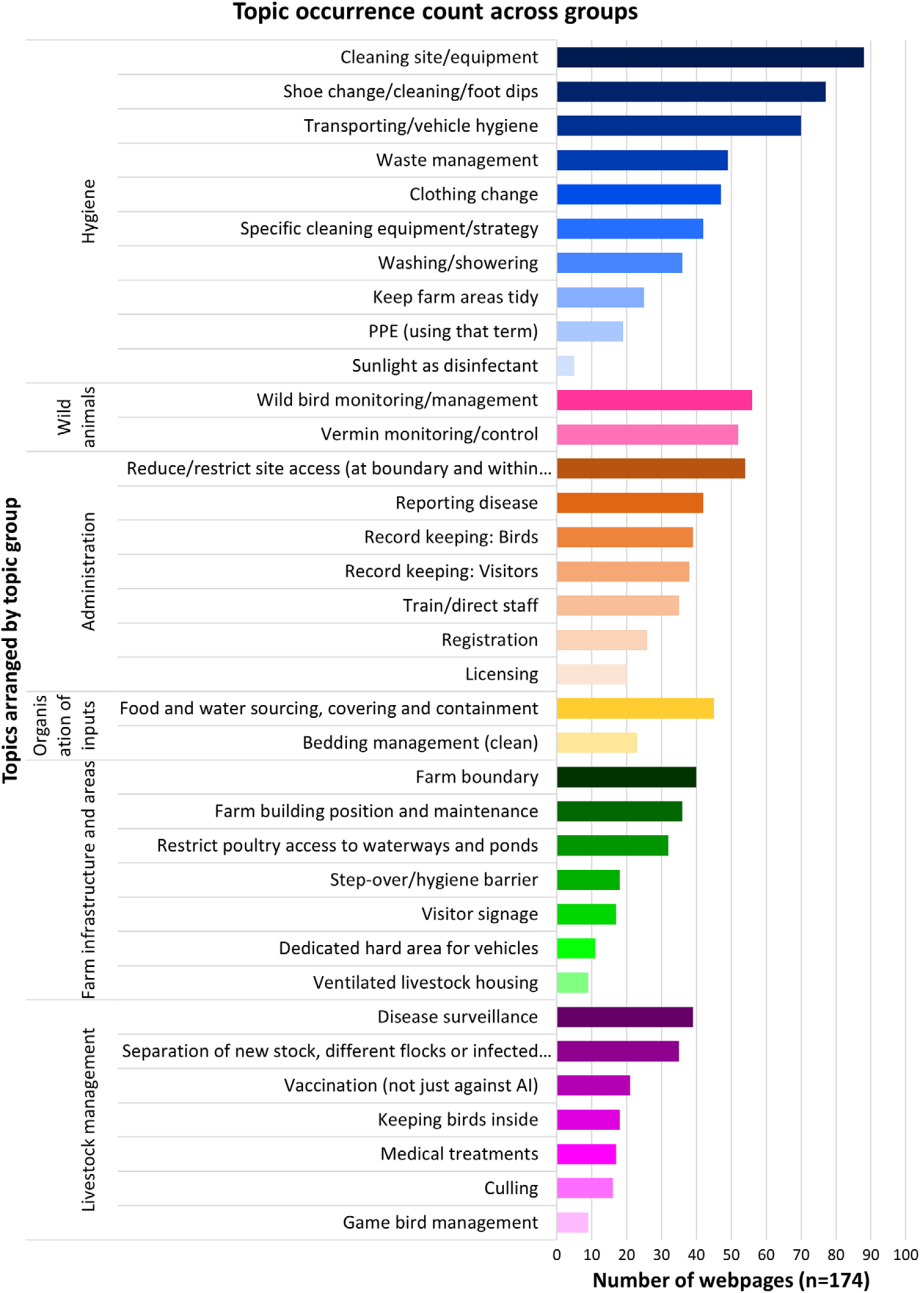
Topic occurrence count across groups.

The most frequently occurring topic from across all the websites was cleaning of farm site/equipment (*n* = 89), followed by footwear change/cleaning/foot dips (*n* = 77) and transport/vehicle hygiene (*n* = 71). The next most frequently occurring topic was from the wild animal category: wild bird monitoring/management (*n* = 56) (Figure 4).

Some topics often appeared but only on a subset of stakeholder websites. For example, only five of the 10 websites referenced licensing as part of their biosecurity guidance, three of which were the Welsh, Scottish and UK government websites—license providers and enforcers. Licensing did not feature as a biosecurity topic on webpages from the BVA, BVPA, Red Tractor, BEIC and BPC.

The least frequently occurring topics were on sunlight as a disinfectant (*n* = 5), game bird management (*n* = 9), ventilated housing (*n* = 9) and dedicated hard areas for vehicles (*n* = 11). Notably, these topics often appeared in guidance relating to a specific subset of farms. For example, advice to use sunlight as disinfectant was provided in relation to free‐range farms more than others.

**FIGURE 5:**
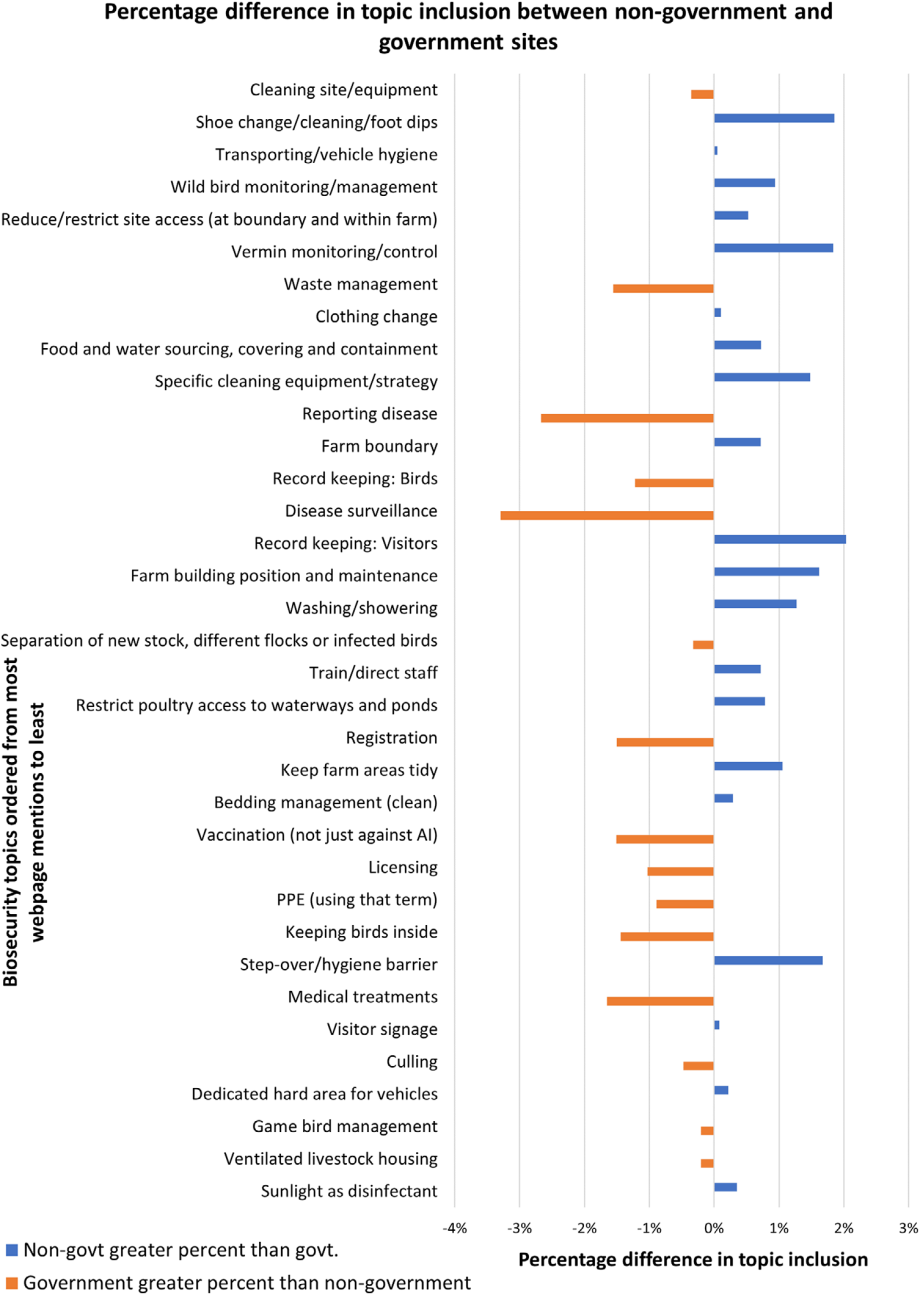
Percentage difference in topic inclusion between non‐government and government sites.

For some of the most frequently included topics, such as cleaning site/equipment and transport/vehicle hygiene, there was no significant difference in emphasis across government or non‐government bodies, reflecting consensus in importance of these topics (Figure 5). However, there are some topics that were proportionally more often discussed on one group's webpages than the other. For example, guidance on disease surveillance, reporting disease and waste management appeared more on government than non‐government websites. For non‐government websites, there were more pages covering topics relating to record keeping: visitors, vermin monitoring/control and footwear change/cleaning/foot dips when compared to government websites.

### Amount of detail

**FIGURE 6:**
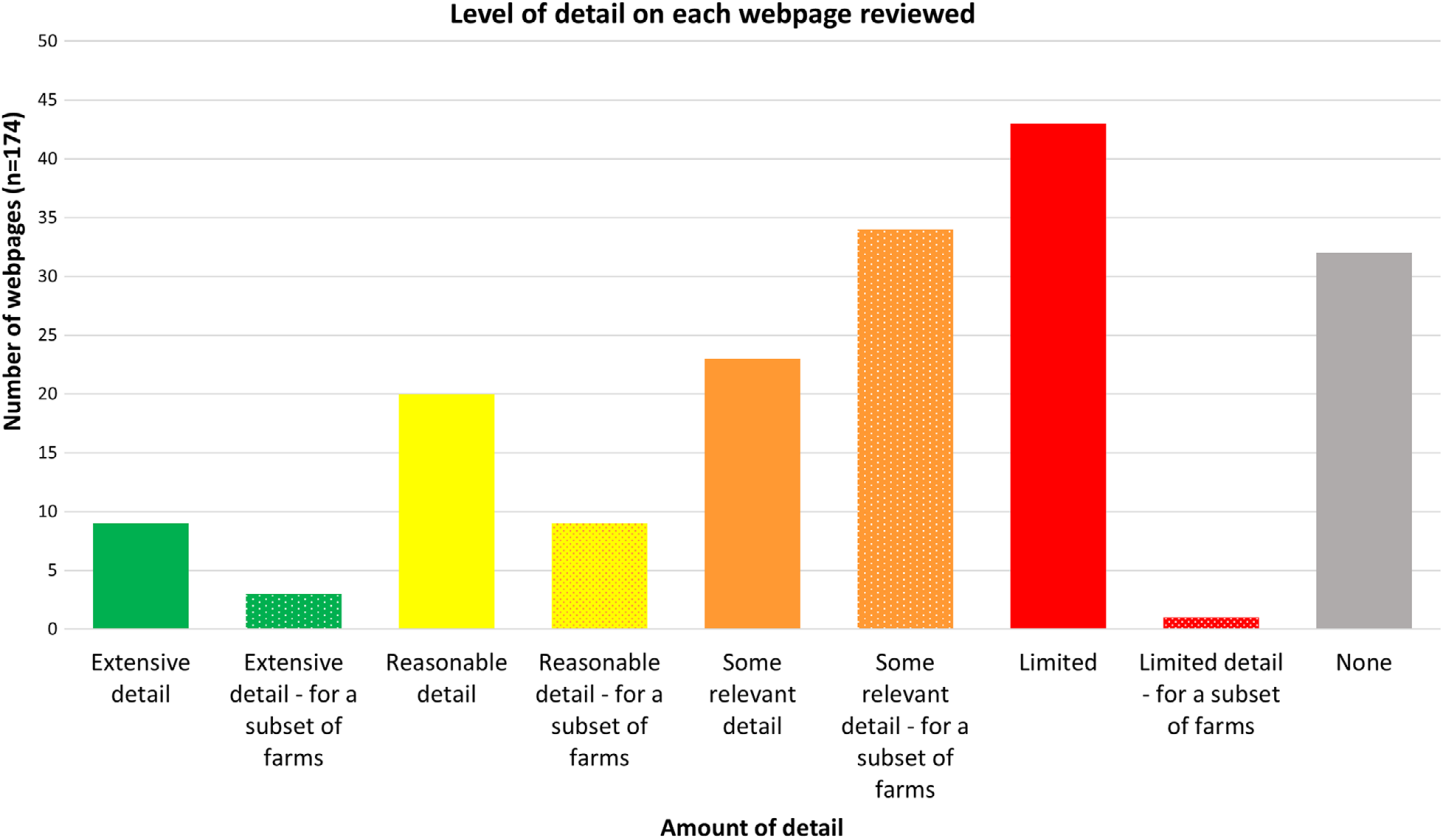
Level of detail on each webpage reviewed.

There were only 12 pages that contained extensive detail (Figure 6). One example was a webpage that appeared in the results of the gov.wales search. It included a Defra document from 2022 titled ‘Biosecurity and preventing welfare impacts in poultry and captive birds’.^9^ Notably, it was not included in the gov.uk results. This 28‐page document included details on what biosecurity does, mandatory housing orders and sections dedicated to a range of biosecurity practices such as deterrents for wild birds. The following excerpt illustrates the level of detail provided. Following a point about using government approved disinfectants in foot dips and a link to further details, it stated:

‘The level of boot dip should always be at least ankle deep and care should be taken to position the boot dip to allow easy step in/step out procedures to take place. Boot dips should be covered to prevent disinfectant from being diluted by rainwater or inactivated by ultraviolet light. It may be preferable to have specific footwear that is only worn in the bird area, changing footwear upon entering and leaving the area accessed by birds’.^9^

Webpages containing this amount of detail were a rarity. Instead, most contained either limited details (*n* = 43), some relevant detail—for a subset of farms (*n* = 34) or no details (none; *n* = 32) (see Table 3 for definitions). As one example of how relevant but limited details might appear, a BPC webpage titled ‘A guide to interventions’ focusing on biosecurity to prevent Campylobacter stated the following about footwear changes and foot dips:

**TABLE 3:** Specific definitions used to assign a level of detail against each webpage in a consistent way.

Amount of relevant guidance for poultry keepers	Definition
None	The webpage may or may not explicitly refer to biosecurity. It provides no description of biosecurity and no guidance on how to implement it. It may contain relevant onward links but no direct guidance.
Limited	The webpage explicitly refers to biosecurity. It provides some description of what biosecurity might include but provides no specifics on how to implement biosecurity using generalities only, for example, monitor your birds for disease.
Some relevant details—for a subset of farms	The webpage explicitly refers to biosecurity and provides some guidance directed at a subset of farms (i.e., layer, broiler, grandparent). It contains general mention of biosecurity measures and at least one specific piece of guidance on how a biosecurity measure might be implemented.
Some relevant details	The webpage explicitly refers to biosecurity and provides some guidance. It contains general mention of biosecurity measures and at least one specific piece of guidance on how a biosecurity measure might be implemented.
Reasonable detail—for a subset of farms	The webpage explicitly refers to biosecurity and provides multiple descriptions of where a subset of farms should be applying biosecurity. It contains multiple specific examples or guidance details regarding implementation.
Reasonable detail	The webpage explicitly refers to biosecurity and provides multiple descriptions of where farms should be applying biosecurity. It contains multiple specific examples or guidance details regarding implementation.
Extensive detail—for a subset of farms	The webpage explicitly refers to biosecurity and goes into detail about one or multiple biosecurity measures directed at a subset of farms. It contains one or more detailed descriptions of how biosecurity measures can be applied effectively. It includes details on the purpose of at least one biosecurity measure.
Extensive detail	The webpage explicitly refers to biosecurity and goes into detail about one or multiple biosecurity measures. It contains one or more detailed descriptions of how biosecurity measures can be applied effectively. It includes details on the purpose of at least one biosecurity measure.

**TABLE 4:** Source websites and number of webpages reviewed within each site following search and narrowing for relevance process.

Reviewed webpage overview				
Name of stakeholder	Source of data (website)	Type of body	Original search results from ‘biosecurity poultry’	Webpages included in final analysis including onward links
UK Government	www.gov.uk	Government	17,433	46
Scottish Government	www.gov.scot	Government	1068	34
Welsh Government	www.gov.wales	Government	239	32
British Free Range Egg Producers Association	www.bfrepa.co.uk	Quality assurers	NA	22
National Farmers’ Union	www.nfuonline.com	Union	50	16
British Poultry Council	www.britishpoultry.org.uk	Sector advocates	34	10
Red Tractor and Red Tractor Assurance	www.redtractorassurance.org.uk/www.redtractor.org.uk	Quality assurers	44	5
British Veterinary Poultry Association	www.bvpa.co.uk	Veterinary sector advocates	NA	5
British Veterinary Association	www.bva.co.uk	Veterinary sector advocates	160	3
British Egg Industry Council—including British Lion	www.britisheggindustrycouncil.com	Sector advocates	NA	1

*Note*: ‘NA’ site figures indicate sites that were manually searched and lacking search function.

**TABLE 5:** Number of webpages with clear dates of publication and/or date of most recent update across each website.

Webpage publication date summary			
Webpage	Webpages with dates	Webpages about avian influenza with dates	Undated webpages
UK Government (*n* = 46)	36	21	10
Scottish Government (*n* = 34)	30	11	4
Welsh Government (*n* = 32)	29	12	3
BFREPA (*n* = 22)	20	6	2
NFU (*n* = 16)	5	3	11
BPC (*n* = 10)	10	7	0
BVPA (*n* = 5)	0	0	5
Red Tractor Assurance (*n* = 5)	0	0	5
BVA (*n* = 3)	2	1	1
BEIC (*n* = 1)	1	0	0

Abbreviations: BEIC, British Egg Industry Council; BFREPA, British Free Range Egg Producers Association; BPC, British Poultry Council; BVA, British Veterinary Association; BVPA, British Veterinary Poultry Association; NFU, National Farmers’ Union.

‘Good husbandry requires farmers to enter the houses a number of times every day to assess the health and welfare of birds, so consistent application of disinfectant foot dips, changing boots and clothing, and physical barriers to stop dirt entering from outside are important elements’.^10^

While the key message about using foot dips and encouraging boot changes were present, the practicalities of those interventions were absent. This was often the case, with key messages shared frequently but briefly across webpages.

**FIGURE 7:**
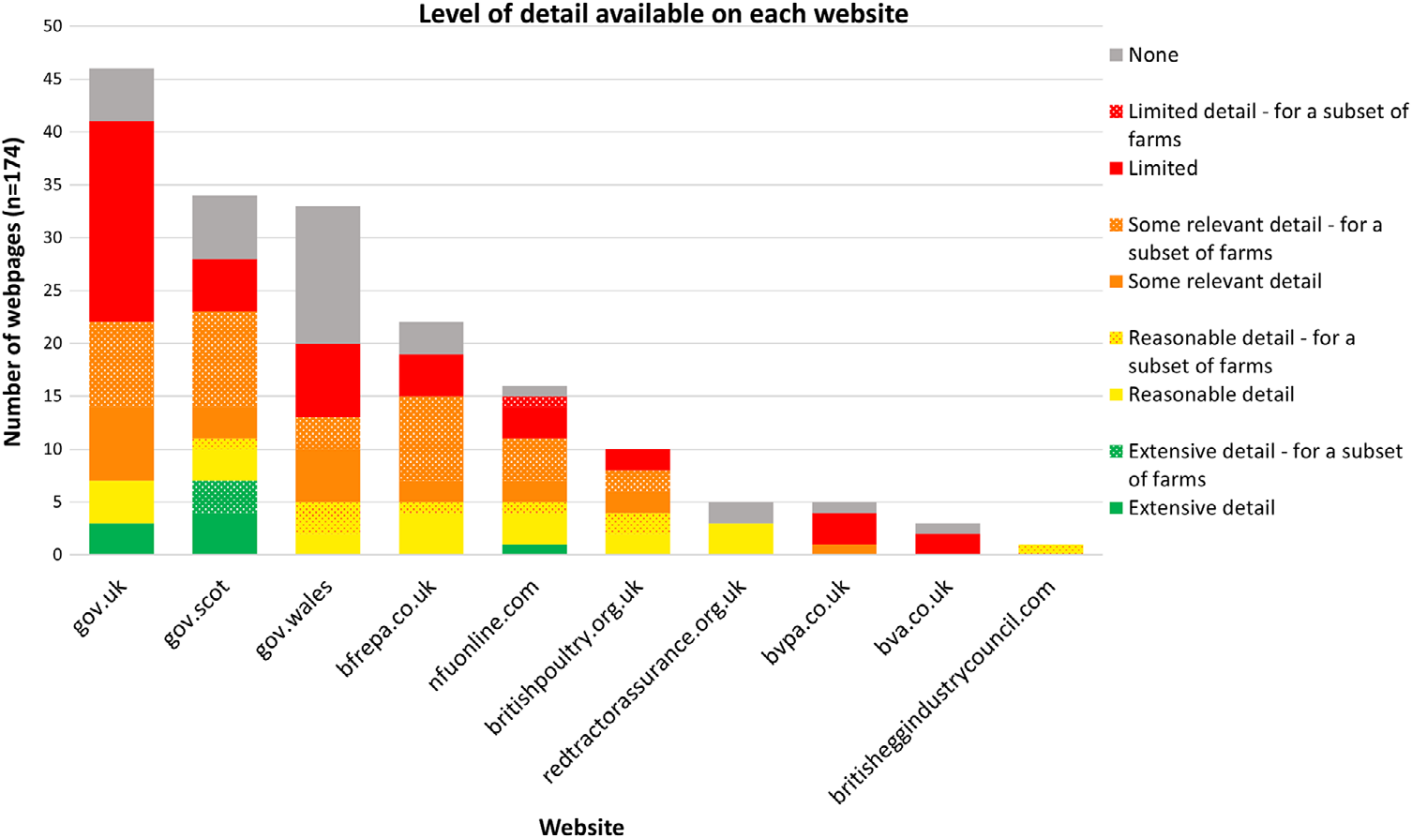
Level of detail available on each website.

The website with the greatest number of webpages with reasonable or extensive detail was gov.scot (*n* = 11, 32%) (Figure 7). Relative to the number of relevant pages they hosted overall, the BPC and Red Tractor Assurance had the largest proportion of webpages with reasonable or extensive details (40% and 60%, respectively).

Overall, gov.uk had the largest number of pages with detail categorised as limited or none (*n* = 24, 52%). While gov.uk webpages regularly contained multiple onward links, only five (20%) of the webpages with limited or no details linked through to any of gov.uk's three most informative pages with extensive detail.

**FIGURE 8:**
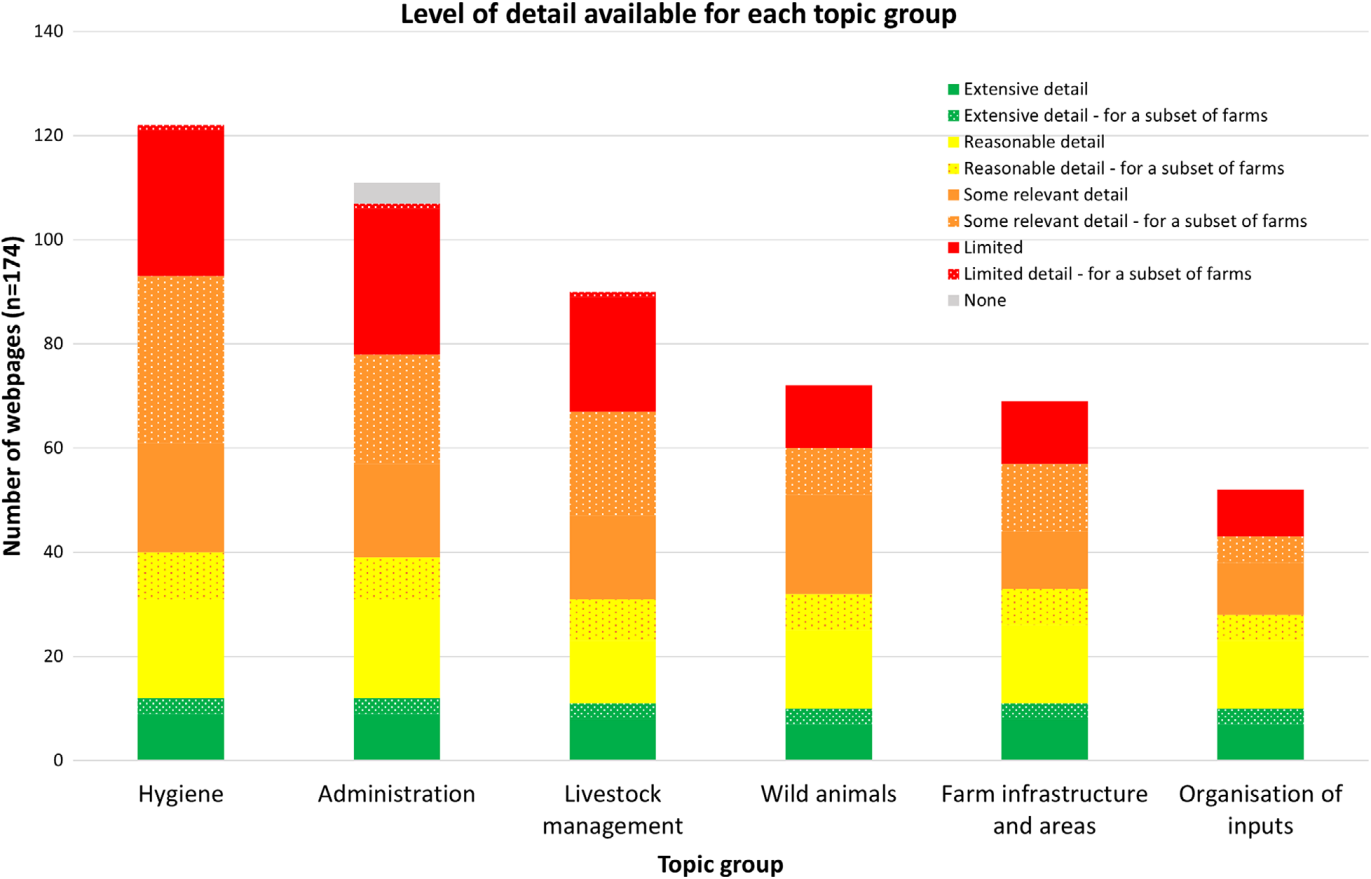
Level of detail available for each topic group.

The number of webpages containing extensive or reasonable levels of detail was similar across the six topic groups. The number of webpages overall for each topic group is what differed, with some covered more often but in less detail than others (Figure 8).

### Pathways to reasonable and extensive detail

**FIGURE 9:**
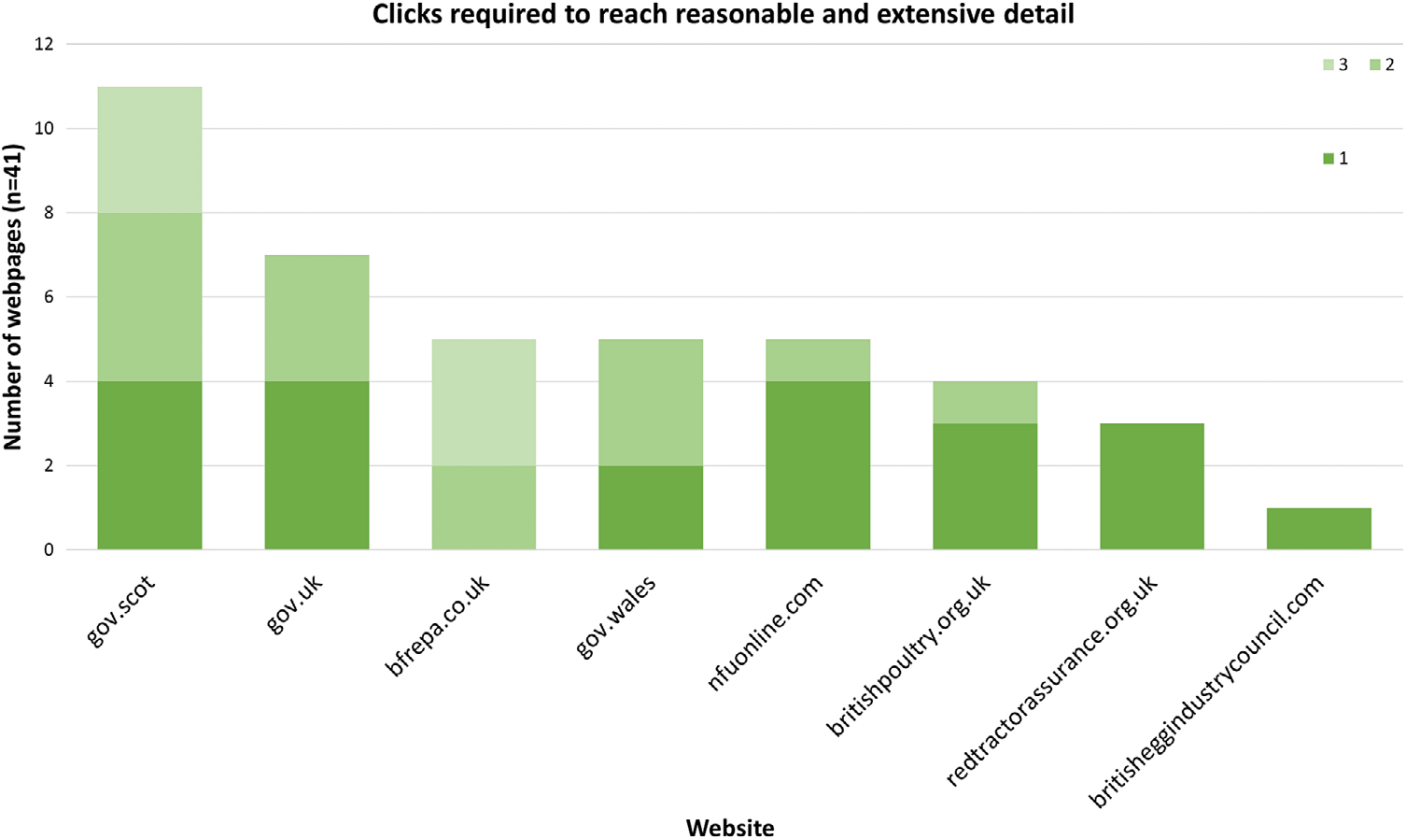
Clicks required to reach reasonable and extensive detail.

The review captured the clicks it took to access each webpage, with one meaning the links appeared within search results, and anything more accessed via links within those webpages (Figure 9). gov.scot had the largest number of webpages with reasonable or extensive detail (*n* = 11), all requiring between one and three clicks to reach. While gov.uk had fewer detailed pages (*n* = 7), all were accessible within one or two clicks, similar to gov.wales. The NFU's website (nfuonline.com) had the largest proportion of content with reasonable and extensive detail, with 80% (*n* = 4) of those pages accessible immediately from the search results.

**FIGURE 10:**
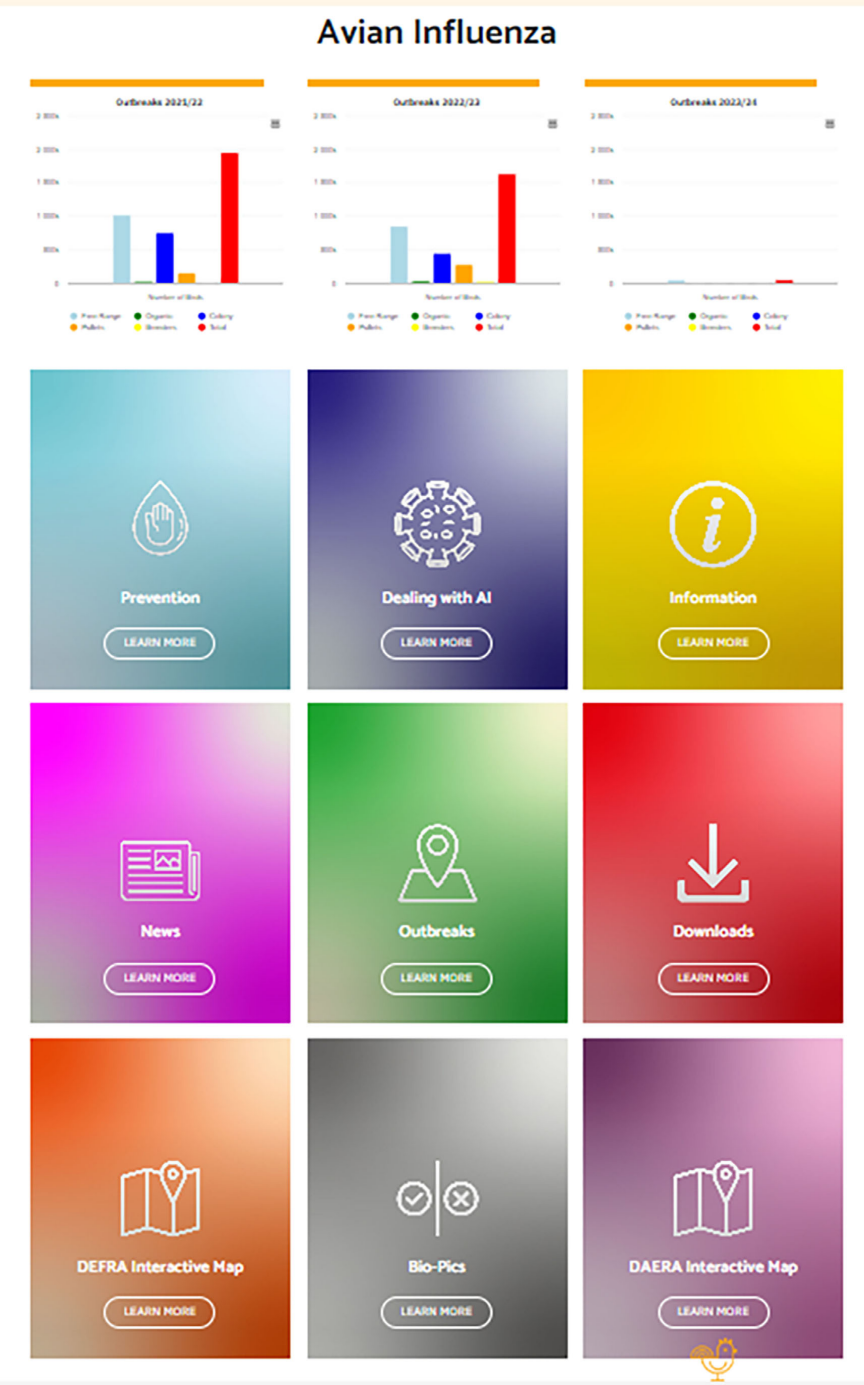
Screenshot from British Free Range Egg Producers Association's website of avian influenza navigation page.

The detailed webpages that took three clicks to get to from gov.scot were older (dating from 2002, 2003 and 2007) and discussed *Salmonella* prevention among broiler, breeding and laying flocks. Notably, gov.scot was the only website where *Salmonella* guidance came up in the search results for biosecurity information. The remaining webpages with detailed information that required three clicks were from BFREPA. In general, navigating this site proved difficult as webpages about AI were accessed via a dashboard of nine links—the contents of which were not always apparent from link titles (Figure 10). Onward links via lists and dashboards of this kind channel the user through the website until they reach a (hopefully) relevant webpage.

Unlike BFREPA, gov.uk used longer, more general pages to direct towards specific information. One example—titled ‘Bird flu (AI): how to prevent it and stop it spreading’^11^—was a single, lengthy page containing a range of topics. The first part of the page was a hyperlinked content lists, allowing the reader to jump to the most relevant topic while remaining on the same webpage. Each section provided key messages, and some contained detailed instructions. Others contained links to additional information. For example, the section on cleaning links to a page about Defra‐approved disinfectants.

### Conflicting advice

While the stakeholder websites tended to cover the same topics, advice on how to implement biosecurity measures relating to those topics was not always consistent. For example, in the case of footwear covers.

**FIGURE 11:**
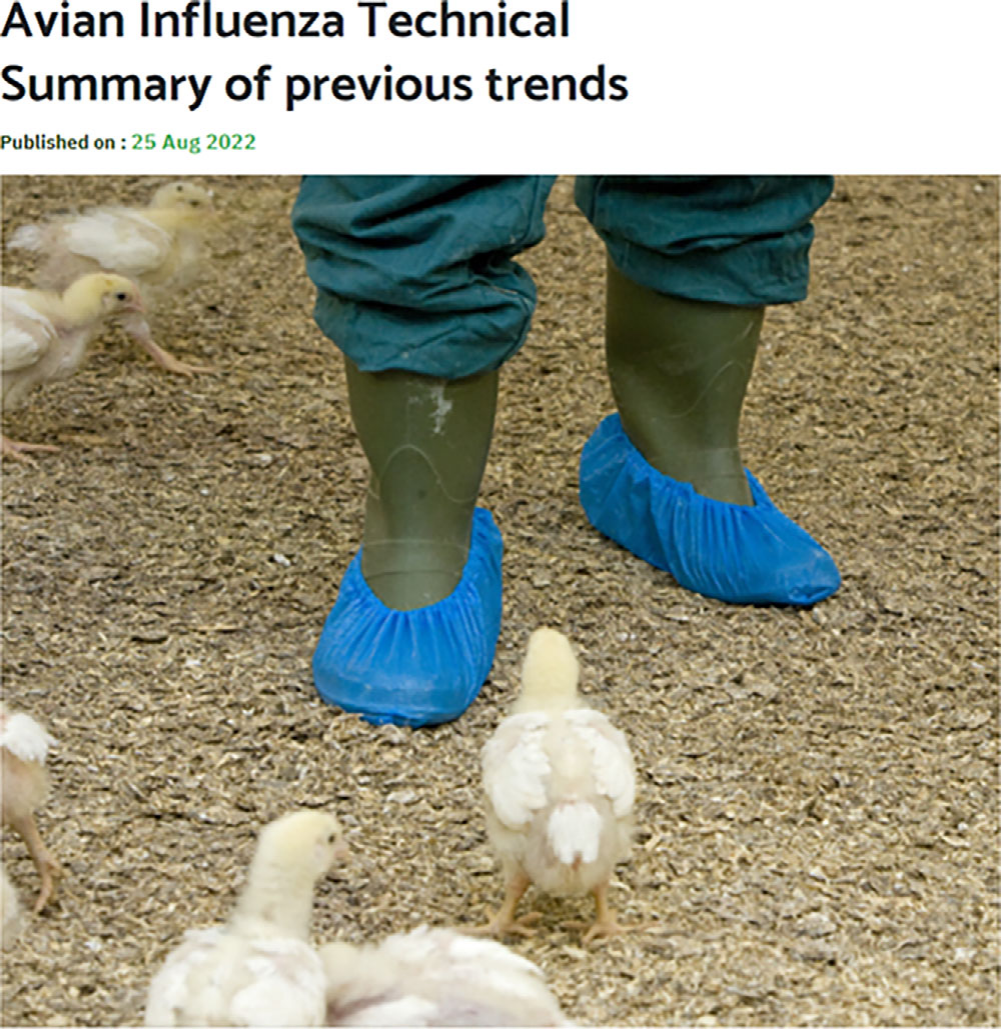
Screenshot from British Free Range Egg Producers Association's website showing use of footwear covers.

Of the webpages reviewed, 77 included guidance relating to footwear change/cleaning/foot dips. While most promoted cleaning footwear or changing footwear, some promoted the use of disposable footwear covers. For example, on one webpage from the Scottish Rural College linked to through gov.scot, the guidance suggested the use of these covers with occasional visitors: ‘Supply disposable overalls and disposable foot covers for occasional visitors’.^12^ On BFREPA, a webpage opens with a large image of someone wearing footwear covers (Figure 11).

Other webpages presented shoe covers as less reliable than alternative footwear biosecurity measures. Even within a single website, these messages sometimes coexisted. Despite their inclusion of the image above, BFREPA, for example, said footwear covers (which here they refer to as ‘overboots’) were not effective:

Use of plastic overboots at barrier points is NOT recommended. Plastic overboots tear after a few steps and allow the muck from outside to be introduced into the bird environment.^13^ [Original emphasis]

But on other webpages mentioned the use of footwear covers without concern:

Prior to entering, change footwear to area specific wellies and ensure the physical barrier is used to prevent shared floor space with dirty footwear. Alternatively, place another layer of disposable overshoes onto your footwear.^14^

Mostly, footwear covers were not presented as a detail of biosecurity guidance, with websites instead promoting the use of footwear changes and cleaning as best practice.

## DISCUSSION

There is a limit to legislated requirements relating to poultry farm biosecurity, particularly outside of outbreak periods when additional policies to control outbreaks are not in effect. As a result, most publicly available biosecurity guidance is not linked to external oversight or enforcement (there may be further policies and oversight from companies privately). Instead, webpages present biosecurity measures as important but ultimately the responsibility of poultry farmers. Rather than through law, the importance of many biosecurity measures comes across through the sheer quantity of webpages that discuss them.

This review identified the topics defining biosecurity and that stakeholders promote for UK poultry farms. Topic groups were identified through an iterative process based on the content and information included on each webpage. This means in‐depth analysis of the information provided on each webpage is all that informs how they have been grouped. As such, the groups and topics emerging in this study are broad and, in some instances, overlapping, for example, restricting bird access to waterways focused on farm areas and infrastructure in the guidance provided, but is relevant to risks associated with wild birds that is covered by another topic group. While this study focused on patterns in content to create topics and groups, re‐examining the webpages with predetermined categories of priority biosecurity information and key messages would provide a different structure and potential new insights.

Overall, topics related to hygiene consistently received the most attention, with webpages regularly including reference to multiple hygiene topics on a single page, such as vehicle hygiene, clothing changes and foot dips. The frequency with which these topics were discussed across the websites demonstrates where farmers are told to dedicate their energy when it comes to biosecurity. This consistency in messaging is positive, as it means regardless of what website people look to for guidance, they are likely to receive broadly the same advice. However, not all biosecurity topics are discussed in equal measure. Some webpages emphasised a subset of topics with particular types of farms in mind, for example, where free‐range farms or poultry compartment schemes are the focus, which highlight different cleaning or housing advice in each instance.

If webpages are to be useful, they need to be up‐to‐date and easy to find when needed. This review revealed that some websites have managed to keep information about AI updated within the last 5 years. However, some websites included webpages that had not been updated in over a decade. For example, the BPC had not updated or removed old webpages about AI, adding new webpages alongside older ones. While not inherently bad content, unreviewed older webpages can impact the accuracy and consistency of guidance available, along with the navigability of websites. Furthermore, the shift in the major incursion threat from other poultry to wild birds, since 2021, means that the emphasis on different biosecurity measures on farms should have shifted online.

Across the websites reviewed, the number of webpages available raises questions about how easy it is to navigate to the level of detail farmers require. As seen on sites such as gov.uk, lots of pages might be available and only a small number link through to the site's most detailed pages. While it may be that the most extensively detailed pages are not always the most relevant link to be shared on every page, it does reflect that a user's entry point to the site and topic greatly informs whether they will be able to access relevant and detailed information. Ironically, the biggest challenge can be finding information about the topics that are discussed most. For example, while topics such as cleaning the site/equipment and footwear change/cleaning/foot dips were often mentioned, in most cases, guidance was not very detailed. Posters are the best example of this, containing messages such as ‘Implement effective vermin control’ but lacking guidance on how to go about that. Having limited detail is not inherently bad—as short memorable or eye‐catching messages are likely intentionally circulated and easier to get someone to engage with than a lengthy set of instructions. Equally, some of these webpages may intend to circulate key messages but rely on other sources to provide specifics. However, it is important to ensure that when people want detailed guidance that information is available and clearly signposted. If stakeholders think that readers should seek detail from other external sources, those sources should be made explicit on their websites. The BPC's page ‘A guide to interventions’ provides one example of a lengthy webpage that appears it would contain useful specifics but falls short.^9^

Lack of detail can also limit understanding of why certain measures are important. For example, the emphasis on administrative tasks that included topics such as record keeping: birds and licensing was quite different from other biosecurity guidance. Government websites placed particular emphasis on these administrative areas and promoted reporting and surveillance measures more than non‐government websites. This demonstrates the importance that government agencies placed on traceability of outbreaks and their responsibility for outbreak response on a national scale. Rather than preventing disease, this administrative guidance promoted data gathering for use should disease occur—namely, for contacting relevant parties, figuring out where disease incursions could have come from and noticing disease should it arrive within the flock. This guidance also included templates for visitor records that illustrated what should be captured and facilitate record keeping. However, very little information was provided explaining the value of these administrative tasks. Therefore, their inclusion on pages focusing on biosecurity could seem disconnected for a reader focused on preventing disease. There are other topics that are part of a more holistic approach to biosecurity, such as ventilation in poultry housing. Without details of how these additional and less frequently discussed topics link to biosecurity, it is hard for a reader to know whether to implement them.

With many topics mostly discussed in generalities, it is easy to identify a consensus across the sites. However, when looking at the details where they are provided, some differences in priorities and messaging do emerge. While messages do not often outright conflict with one another (barring the footwear cover example), they can differ in their emphasis. This makes some websites more relevant for a subset of farms, different periods of outbreak response, or for readers with different levels of existing knowledge.

Notably, links to studies on efficacy regarding any of the biosecurity measures promoted across the sites were entirely absent, with all stakeholders discussing biosecurity in terms of what it should include and sometimes saying what measures achieve, without any references to evidence beyond their own or other stakeholder web content. This means when faced with conflicting information, it is very hard for farmers to determine the best way to proceed.

While there are many avenues through which commercial farmers may be accessing information (e.g., via veterinarians, paid for/member‐only webpages, in‐person training or social media) this study provides initial insights by focusing on publicly available webpages. This allows close examination of the topics and content of guidance shared publicly by key stakeholders. However, further research is needed to explore the use of these sites in practice alongside other information sources. Studies exploring the efficacy of human and animal health messaging and education efforts across a variety of countries, farm types and health concerns have revealed the relevance of multiple information pathways both online and in‐person.^15^, ^16^, ^17^, ^18^ The efficacy of each communication method differs based on the context. Thus, websites are just one source, and further work is needed to understand the significance of their role in biosecurity messaging among commercial poultry farmers in the UK specifically.

## CONCLUSION

Overall, there is good availability of pages promoting biosecurity and providing guidance for poultry farmers. Many of the pages broadly agree on priority topics, including cleaning and disinfection of the farm site and equipment; footwear and vehicle hygiene; and the need to control wild animal incursions. However, specific guidance on how to implement these suggestions is either lacking or difficult to navigate to. No website was found to contain detailed and up‐to‐date instructions regarding how to implement on‐farm biosecurity across more than a subset of key topics. Where one site might excel in providing detail (e.g., on government sites regarding licensing), they would fall short on providing detail on other topics they promoted as vital (such as government websites on vermin control). Thus, for someone seeking comprehensive detail across biosecurity topics, this currently requires multiple websites.

It is positive that pages with the greatest level of detail appeared early in search results and did not involve many onward link clicks to get to. However, discerning which pages were of most use among the large number that exist on pages such as gov.uk created a barrier to information. Therefore, ensuring that there are dedicated pages with extensive details focusing on the priority topics and that these pages are linked to key stakeholder websites would be a valuable improvement. Removing old and extraneous webpages to make the pathways to information clearer would hugely benefit the usefulness of these websites.

If seeking to improve the information and accessibility of biosecurity guidance online, coordination across industry and government would ensure the provision of detailed resources. Linking to those webpages consistently and signposting routes to access detailed content would also guarantee farmers had consistent messages and in‐depth information on topics that need it. This approach has already been applied to address bovine tuberculosis. The website tbhub.co.uk has been created to provide joined up advice between government, agricultural and veterinary organisations with this same agenda in mind, and thus could be used as a template upon which an AI hub could be based.

This research has identified what existing stakeholder websites contain and highlighted some areas to improve. Now, to fully understand how improvements can be made, it would be valuable to explore whether farmers use any of these websites and how their use combines with biosecurity guidance from other sources. There are many actors involved in the poultry sector whose roles are not represented through websites—for example, veterinarians—who may play a prominent role in providing biosecurity guidance to farmers.^6^, ^7^, ^19^, ^20^, ^21^ However, questions remain about the specifics of influence and information networks in the UK poultry sector, as well as the consistency of information that veterinarians share within it.^22^, ^23^ The substance and influence of information shared between farmers and via social media should also be investigated. Combining this contextual understanding with rigorous content reviews would help determine how biosecurity guidelines can be improved.

## AUTHOR CONTRIBUTIONS

Eve Houghton, Ivo Syndicus, Pablo Alarcon, James Wood and Paniz Hosseini contributed to the study design. The scoping review was carried out by Eve Houghton. The analysis was conducted by Eve Houghton and Timothy Borthwick. Eve Houghton drafted the initial manuscript, and all the authors contributed to and approved the final paper.

## CONFLICT OF INTEREST STATEMENT

The authors declare they have no conflicts of interest that could be perceived as prejudicing the impartiality of the research reported.

## ETHICAL STATEMENT

This research involved the review of information from publicly available websites. No identifiable information of indivuduals was present on the sites or included in this study. As such, through self‐assessment researchers determined this review did not require additional ethical approval.

## Supporting information



Supporting Information

## Data Availability

The data that support the findings of this study are available from the corresponding author upon reasonable request.
