# Supplementary material for: Scoping review to assess online guidance about poultry farm biosecurity for farmers in the UK
Source: Vet Rec. 2025 Dec 4;198(4):e127–41. doi: 10.1002/vetr.5775 (PMC12904082; doi:10.1002/vetr.5775)
Supplement: Supplementary file 1 — Supporting Information [file VETR-198--s001.pdf]

Supplementary Information

Data extracted from/assigned to every webpage reviewed

Data type	Description
Source level	Numbered 1-3 based on whether the webpage appeared in initial search results (1) was housed within a link on a page from the results (2) or was housed three link clicks on from search results.
Source	Stakeholder website URL on which search was conducted
Category of source	Categorised as government or non-government
Source specifics	Webpages published be specific departments or sub-groups within an stakeholder website were noted (e.g. APHA, Defra)
Content type	Webpage content was classified as guidance; regulation/policy/order; press release; poster; license; collection or form.
Date of access	Date when webpage was first catalogued and accessed
Date of publication	Date the page reports being published (where available)
Date of most recent update	Date the page reports being most recently updated (where available)
Search terms	For level 1 sources found through initial search, the search term 'Biosecurity poultry' or 'biosecurity' was recorded
Link	URL to each webpage
Title	Webpage title
Bird/farm specifics	If the page related to specific groups of livestock (e.g. poultry) or farm types (e.g. duck or layer)
Specifically about Avian Influenza?	Noted if the webpage specifically addresses the topic of avian influenza at all
Topics covered	Note of topics discussed in relation to biosecurity
Period of focus	If page had a focus on prevention, outbreaks or post-outbreak periods
Key relevant message(s)	Noted any messages given particular focus (indicated by greater quantity of content)
Level of biosecurity guidance relevant to poultry farmers	*See Table 2 in manuscript*
Proof of position: Evidence/references/citations	Noted if advice was ever supported with additional evidence or citations
Links on to relevant specifics?	Noted if that page contained additional links to provide more detail on topics discussed (could be duplicates of other pages reviewed)
Links for further analysis (not duplicates)	Record of additional links to include in review as level 2 or 3 sources – excluding any links that would duplicate existing search results

Topic groups and topics relating to biosecurity captured for every webpage

Topic group	Group definition	Topic	Topic definition
Farm admin	Any topic that involves paperwork as the main activity such as record keeping, licensing and farm registration. This theme also includes people management as a step associated with biosecurity, such as training staff.	Licensing	Page specifies the need for licenses as biosecurity measure and guidance on how to apply for them e.g. license to move poultry when in a Avian Influenza Prevention Zone
		Record keeping: Birds	Page suggests that keeping records about the poultry on the farm is relevant to biosecurity, including production records; mortality; water and feed consumption; medicine use
		Record keeping: Visitors	Page suggests that farms should register with the government as part of good biosecurity practice
		Reduce/restrict site access (at boundary and within farm)	Page suggests that access to the farm and specific buildings within it should be limited (no mention of physical interventions)
		Registration	Page suggests the need for farms to report signs of disease (to vets, APHA or other government links)
		Reporting disease	Page promotes staff training as an aspect of attaining farm biosecurity
		Train/direct staff	Page suggests that a record of visitors should be kept, which may include recording vehicle entry
Farm infrastructure and areas	Any topic that involves physical buildings or spatial delineation of areas within the farm or signifying its boundary.	Dedicated hard area for vehicles	Page suggests that vehicles should have hard area for parking or cleaning
		Farm boundary	Page suggests that biosecurity efforts should include fencing or a clear division between on-farm and off-farm areas with a physical boundary or entry
		Farm building position and maintenance	Page suggests maintaining farm buildings and the importance of where they are located as relevant to biosecurity
		Restrict poultry access to waterways and ponds	Page suggests that farmers should restrict bird access to ponds or waterways through physical containment of birds or fencing/netting of water areas
		Step-over/hygiene barrier	Page suggests that step over or hygiene barriers should be in place at points within the farm

		Ventilated livestock housing	Page suggests that having ventilation is relevant to biosecurity
		Visitor signage	Page suggests having biosecurity signage and site guidance for visitors
Hygiene	Any topic focusing on cleaning, disinfection or practices to maintain hygiene, including across the farm site, farm equipment, vehicles and people.	Cleaning site/equipment	Page suggests *what* should be cleaned and/or disinfected such as the farm, certain areas or equipment
		Clothing change	Page suggests that clothes should be *changed* when entering or moving between buildings on the farm
		Keep farm areas tidy	Page suggests keeping farm areas tidy e.g. removing weeds and not leaving equipment lying around
		PPE (using that term)	Page suggests using Personal Protective Equipment (PPE) as part of biosecurity measures on farms, using the term 'PPE'
		Footwear change/cleaning/footdips	Page suggests footwear management as a biosecurity practice e.g footwear changes, cleaning, boot covers and foot dips
		Specific cleaning equipment/strategy (e.g. scrubbing)	Page suggests *how* cleaning should be performed e.g. promoting the use of particular equipment like a power hose or using a specific approach such as scrubbing
		Sunlight as disinfectant	Page suggests that sunlight can work to disinfect farm items or areas
		Transporting/vehicle hygiene	Page suggests that vehicles within or entering the farm should be cleaned
		Washing/showering	Page suggests the need for human hygiene which may include suggestions for particular washing practices (e.g. hand washing or disinfection) or facilities required for this
		Waste management	Page suggests that waste from the farm (including manure, carcasses, waste water, soiled bedding) should be handled in particular ways
Livestock management	Any topic describing how poultry (or any other animals on the poultry farm) should be handled, kept or managed in relation to disease.	Culling	Page suggests that culling may be involved as part of biosecurity process or response to disease outbreaks
		Disease surveillance	Page suggests that farm managers or workers should look for signs of disease
		Game bird management	Page suggests importance or restrictions relevant to game bird management and disease
		Keeping birds inside	Page suggests that birds should be kept inside as part of biosecurity efforts or rules relating to that when in prevention zones

		Medical treatments	Page suggests that treating birds with medicines is relevant to biosecurity (often not AI related)
		Separation of new stock, different flocks or infected birds	Page suggests that different breeds, flocks, species or infected birds should be kept separately or quarantined
		Vaccination (not just against AI)	Page suggests the use of vaccination
Organisation of inputs	Topics describing input management in relation to biosecurity efforts.	Bedding management (clean)	Page suggests fresh bedding should be handled or stored in a certain way as part of biosecurity effort
		Food and water sourcing, covering and containment	Page suggests specific food and water sourcing or suggests how it should be kept on the farm as part of biosecurity effort
Wild animals	Topics describing need for awareness or specific responses to wild animals in relation to biosecurity efforts	Vermin monitoring/control	Page suggests that controlling vermin including rats and mice should be done as part of biosecurity effort and any suggestions for how to manage them
		Wild bird monitoring/management	Page suggests that wild birds should be considered relevant to biosecurity and any suggestions for how to manage them

Timing focus for content

Period of focus	Definition
Preventative	Publications and guidance suggesting certain actions that mitigate disease risk or improve biosecurity that is not time specific or framed as preventative
During	Guidance framed as relevant for periods of outbreak. This may or may not be linked to an outbreak on a poultry keeper's farm, but might be in periods where Avian Influenza Prevention Zones (AIPZ) are enforced. This code is also applied in relation to information that becomes relevant when disease is suspected, but not necessarily confirmed.
After	Any documents focusing on what farmers should do after they have experienced an outbreak or as a broader outbreak related regulation is removed in the region
